# Supplementary material for: Structural basis for receptor binding and broader interspecies receptor recognition of currently circulating Omicron sub-variants
Source: Nat Commun. 2023 Jul 21;14:4405. doi: 10.1038/s41467-023-39942-z (PMC10362042; doi:10.1038/s41467-023-39942-z)
Supplement: Supplementary file 1 — Supplementary Information [file 41467_2023_39942_MOESM1_ESM.pdf]

Supplementary Information

Structural basis for receptor binding and broader interspecies receptor recognition of currently circulating Omicron sub-variants

Zhennan Zhao et al.

Supplementary Figures 1-9

Supplementary Tables 1-7

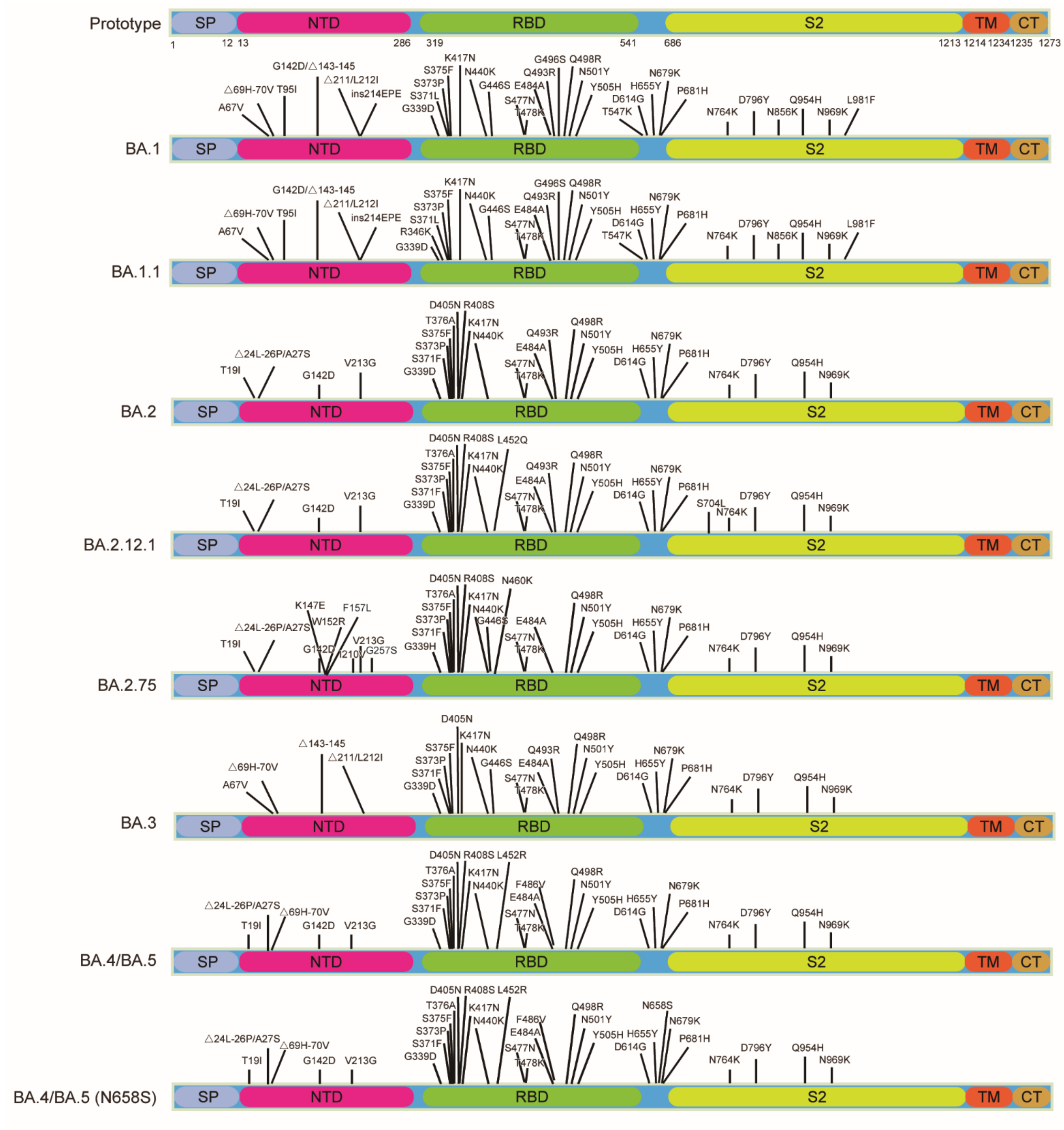

Supplementary Fig. 1 | Schematic diagram for mutation distribution in the S proteins of Omicron sub-variants including BA.1, BA.1.1, BA.2, BA.2.12.1, BA.2.75, BA.3, BA.4/5 and BA.4/5 (N658S).

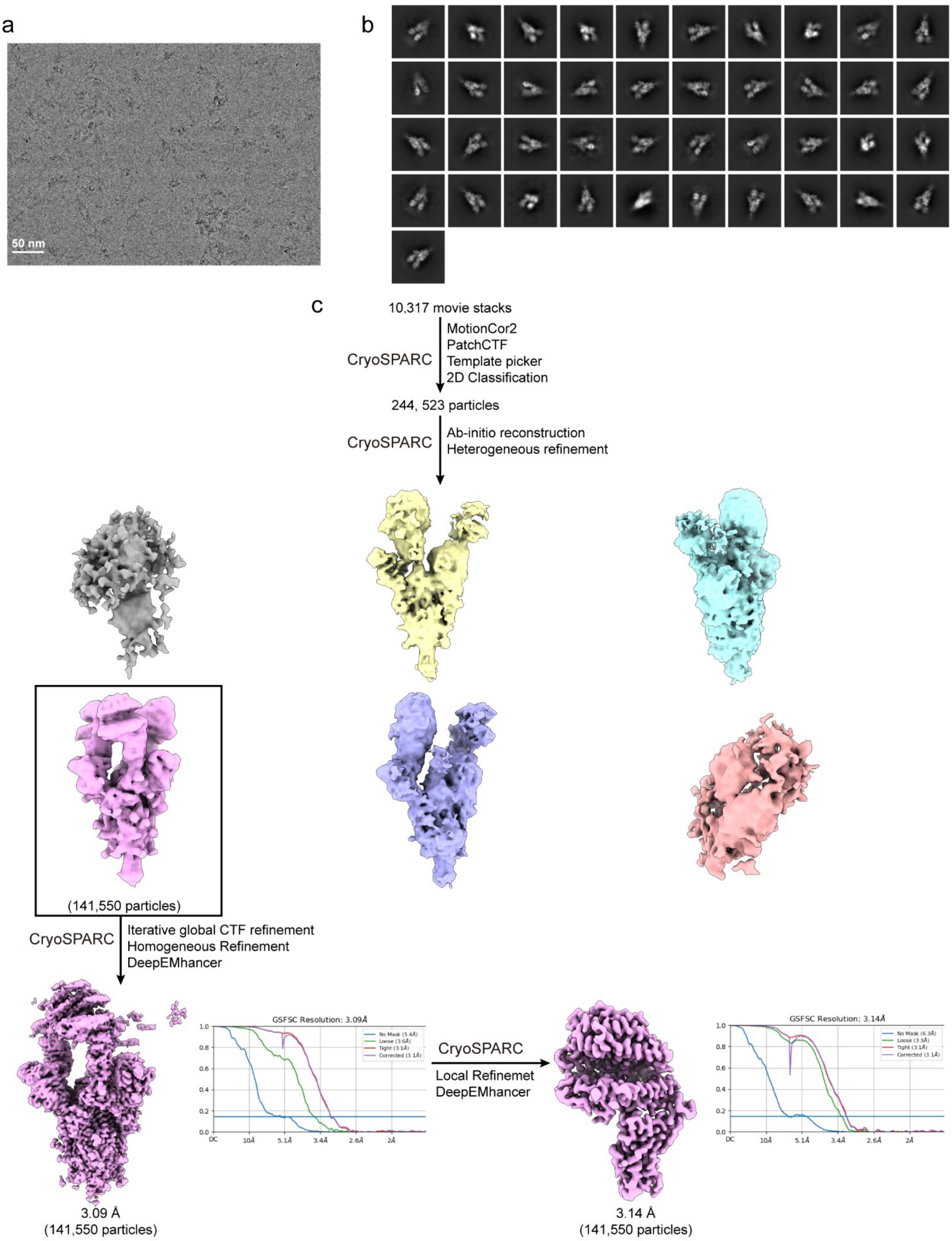

Supplementary Fig. 2 | Flow chart of single-particle analysis of the Omicron BA.2 S/hACE2 complex. a, Representative one from 10,317 cryo-EM micrographs collected for the Omicron BA.2 S/hACE2 complex. **b**, 2D class average images of the Omicron BA.2 S/hACE2 complex. **c**, A workflow of cryo-EM image processing and

reconstruction.

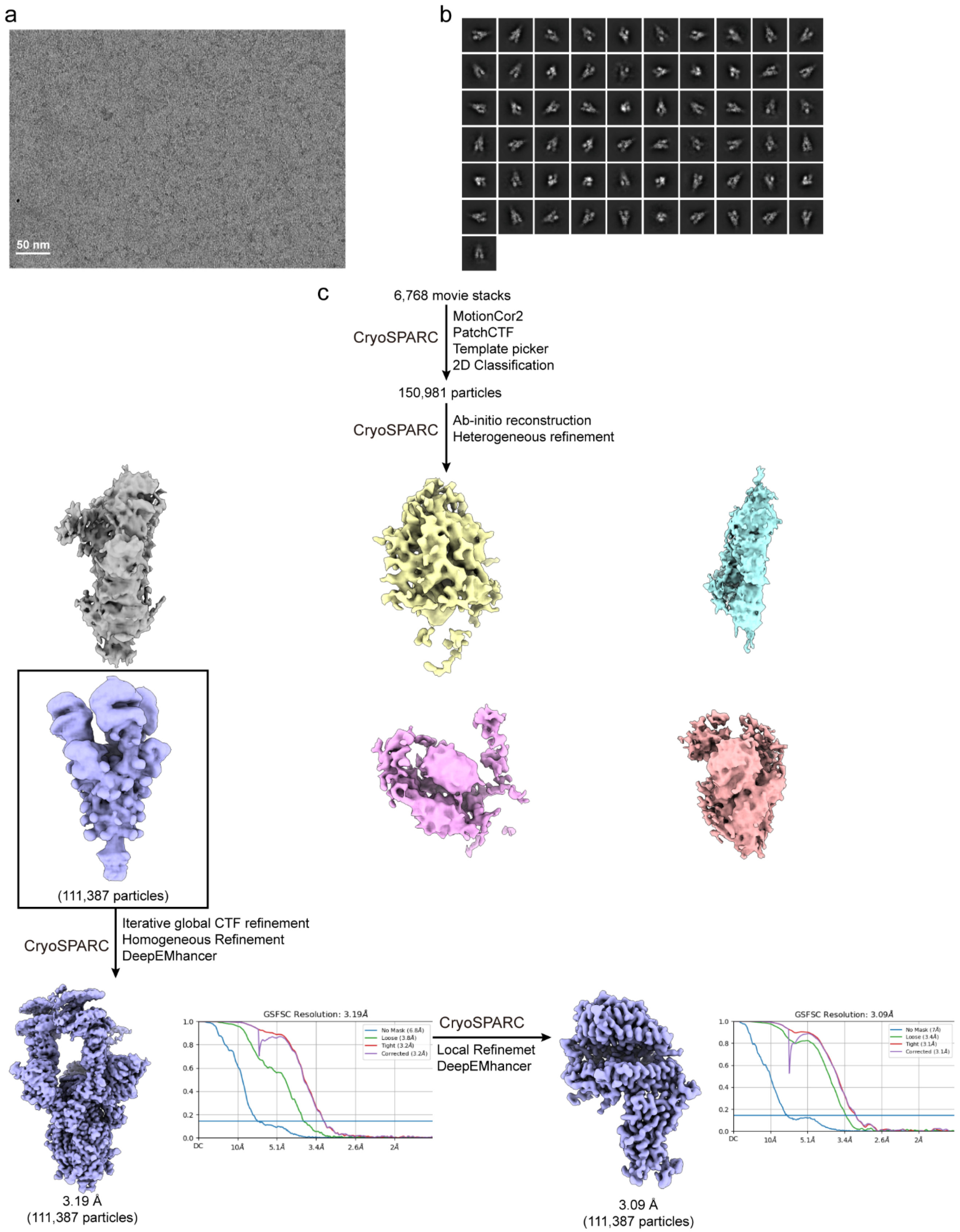

Supplementary Fig. 3 | Flow chart of single-particle analysis of the Omicron BA.2.12.1 S/hACE2 complex. a, Representative one from 6,768 cryo-EM micrographs collected for the Omicron BA.2.12.1 S/hACE2 complex. **b**, 2D class average images of the Omicron BA.2.12.1 S/hACE2 complex. **c**, A workflow of cryo-EM image processing and reconstruction.

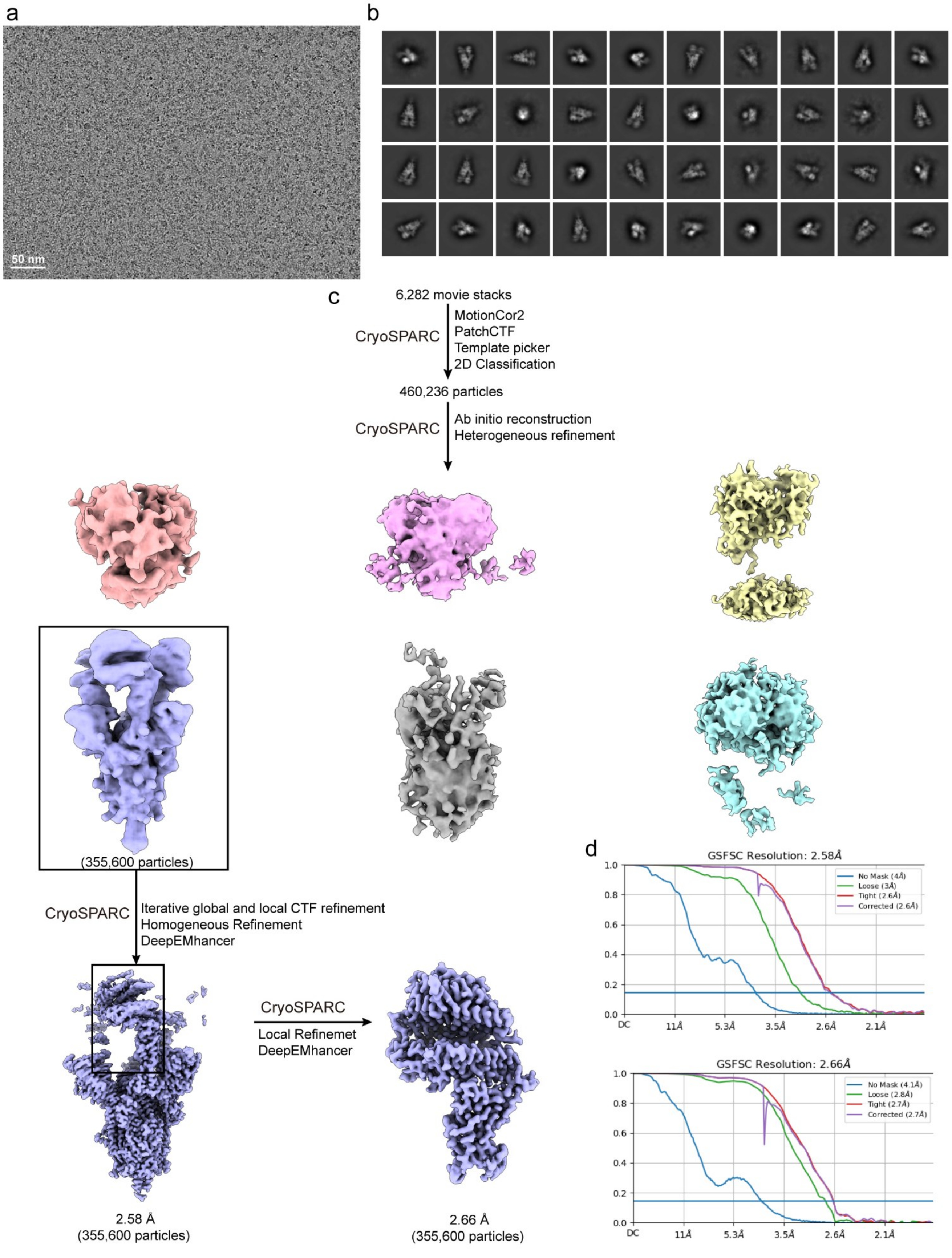

Supplementary Fig. 4 | Flow chart of single-particle analysis of the Omicron BA.4/5 (N658S) S/hACE2 complex.

a, Representative one from 6,282 cryo-EM micrographs collected for the Omicron BA.4/5 S/hACE2 complex. **b**, 2D class average images of the Omicron BA.4/5 S/hACE2 complex. **c**, A workflow of cryo-EM image processing and

reconstruction. **d**, The FSC curve for the density maps.

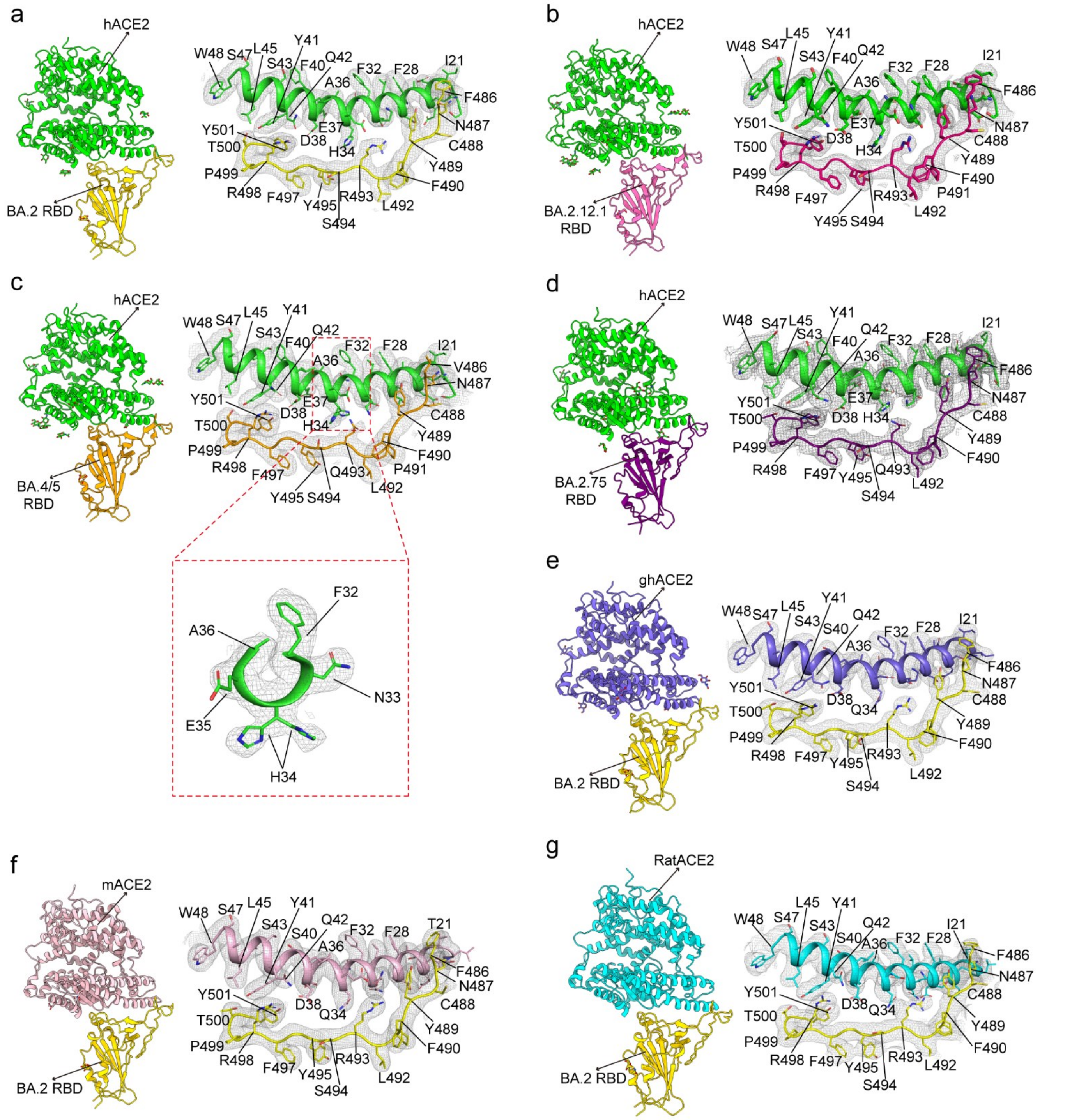

Supplementary Fig. 5 | Local density map of the interface between RBD and hACE2. The Cryo-EM structures of BA.2 (yellow) (**a**), BA.2.12.1 (hot pink) (**b**) and BA.4/5 (orange) (**c**) RBDs in complex with hACE2 (green) were determined by performing local refinement for the BA.2, BA.2.12.1 and BA.4/5 S/hACE2 complexes, respectively. Representative densities on the binding interface were shown as mesh, and AAs were displayed as sticks. The EM density for the two alternative conformations of His34 of hACE2 in the BA.4/5 RBD/hACE2 complex was highlighted in a red dotted box, and this area was zoomed in for visualization. The overall crystal structure of the BA.2.75 RBD (purple) /hACE2 (green) complex (**d**) was shown, and the local 2Fo-Fc map contoured at 0.5σ for its binding interface

was shown as mesh, and AAs were displayed as sticks. The Cryo-EM structures of BA.2 RBD (yellow) in complex with ghACE2 (medium slate blue) (**e**), mACE2 (pink) (**f**) and RatACE2 (cyan) (**g**), which were derived from local refinement, were presented. Their representative densities on the binding interface were shown as mesh, with AAs displayed as sticks.

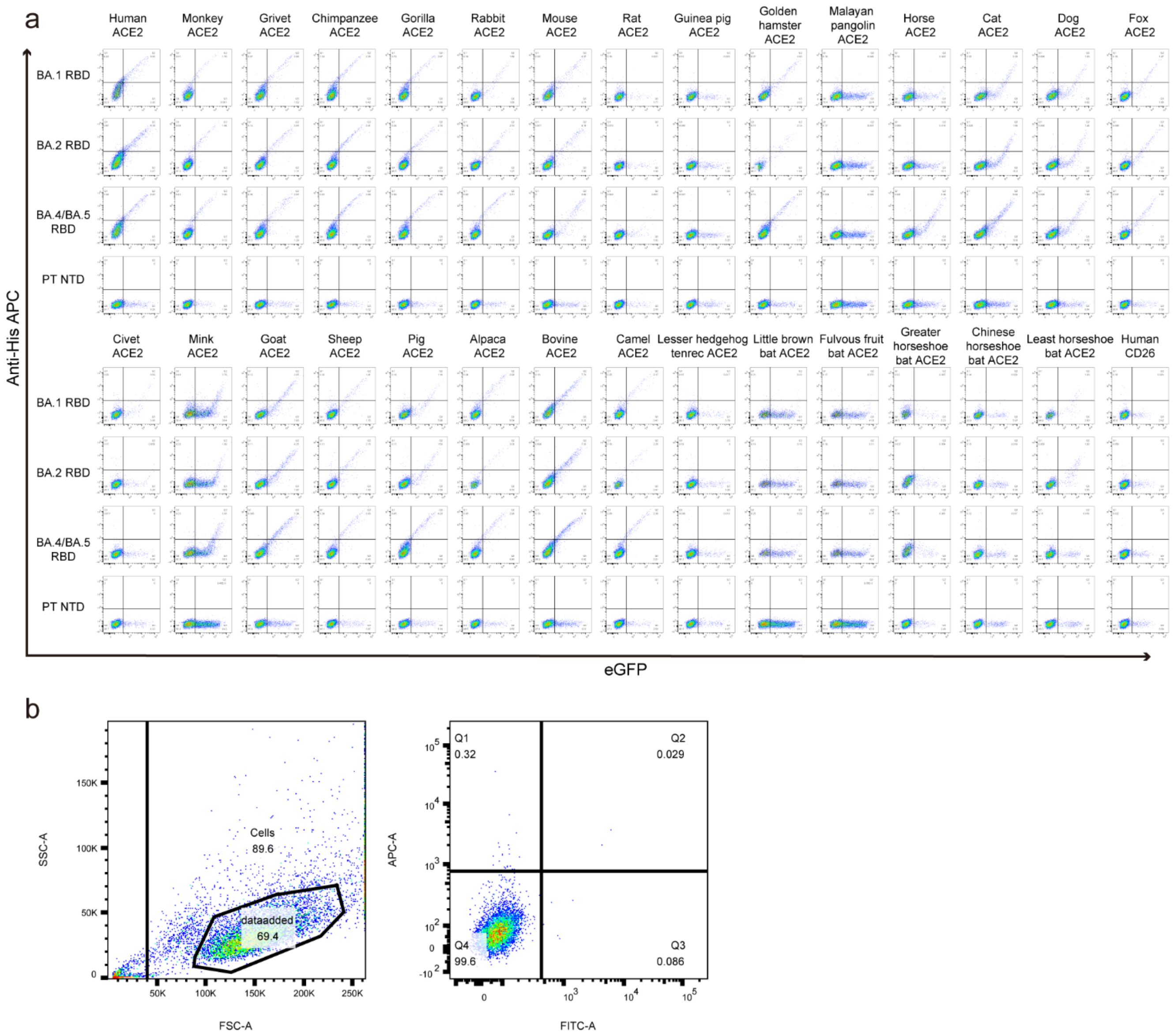

Supplementary Fig. 6 | Evaluation of BA.2 and BA.4/5 receptor binding spectra using FACS. a, One representative for BA.1, BA.2, or BA.4/5 RBD binding human or 28 animal species was shown. The NTD of the prototypic SARS-Cov-2 and human CD26 receptor were used as negative controls. The FACS spectra of BA.1, BA.2, and BA.4/5 RBDs, together with PT NTD, binding to the ACE2 receptor from the indicated species were merged and shown as histograms, as observed in Figure 4. **b**, Gating strategy for FACS analysis of the binding between RBD and ACE2. Live cells were first gated, and untransfected cells were used to define the boundary of positive and negative staining.

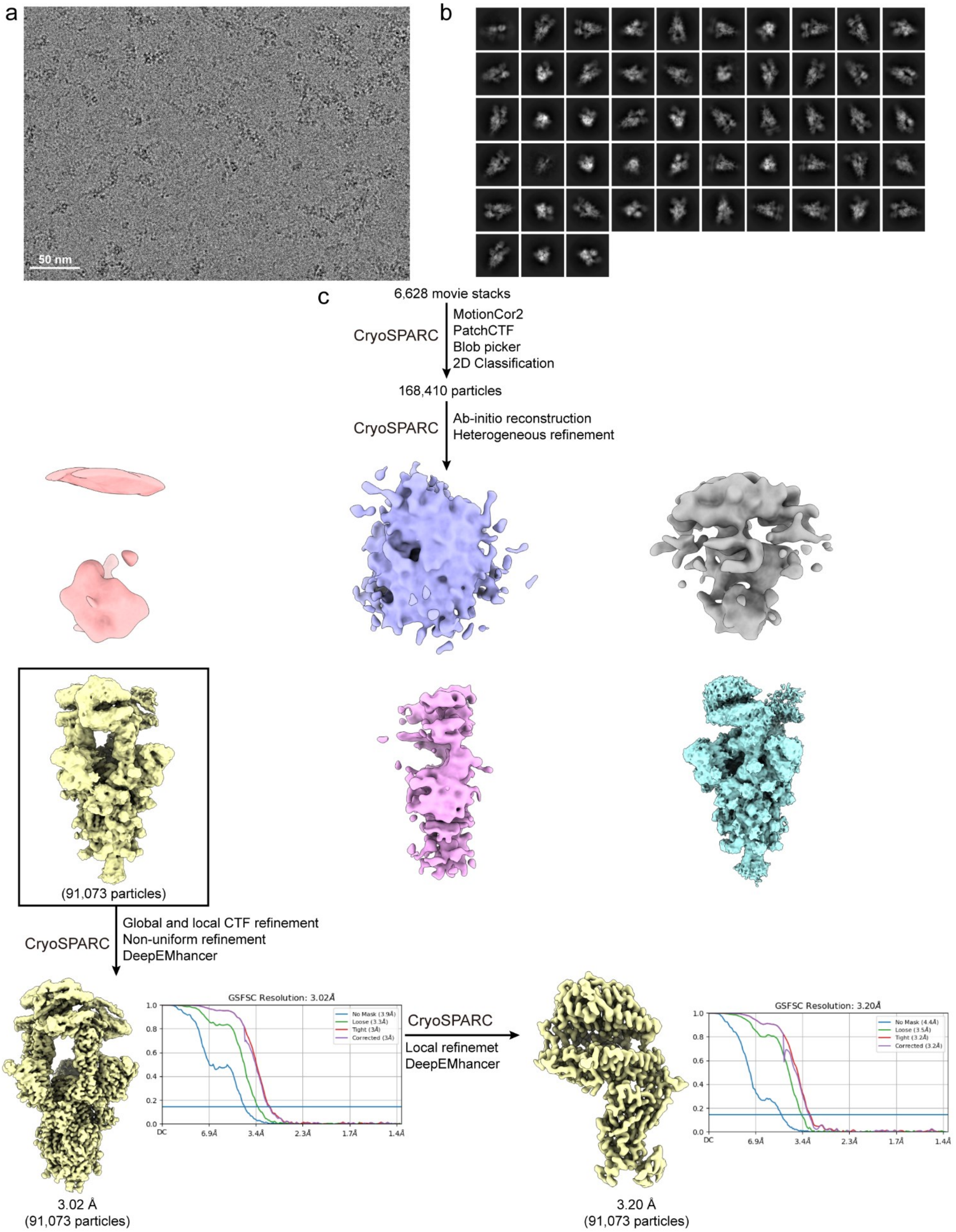

Supplementary Fig. 7 | Flow chart of single-particle analysis of the Omicron BA.2 S/mACE2 complex. a, Representative one from 6,628 cryo-EM micrographs collected for the Omicron BA.2 S/mACE2 complex. **b**, 2D class average images of the Omicron BA.2 S/mACE2 complex. **c**, A workflow of cryo-EM image processing and reconstruction.

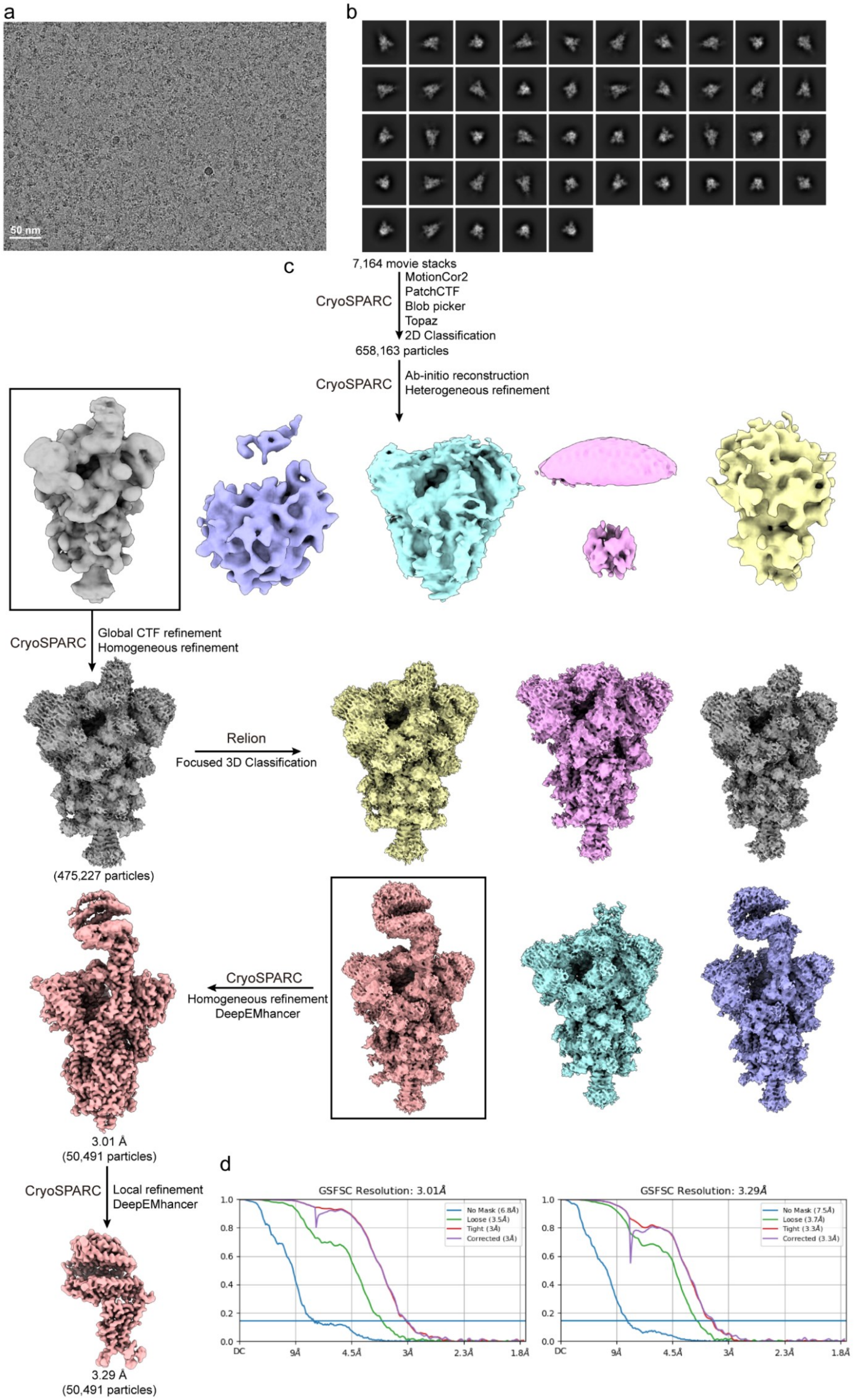

a
50 nm
b
c
7,164 movie stacks
CryoSPARC
MotionCor2
PatchCTF
Blob picker
Topaz
2D Classification
658,163 particles
CryoSPARC
Ab-initio reconstruction
Heterogeneous refinement
CryoSPARC
Global CTF refinement
Homogeneous refinement
Relion
Focused 3D Classification
(475,227 particles)
CryoSPARC
Homogeneous refinement
DeepEMhancer
3.01 Å
(50,491 particles)
CryoSPARC
Local refinement
DeepEMhancer
3.29 Å
(50,491 particles)
d
GSFSC Resolution: 3.01Å
No Mask (6.8Å)
Loose (3.5Å)
Tight (3Å)
Corrected (3Å)
GSFSC Resolution: 3.29Å
No Mask (7.5Å)
Loose (3.7Å)
Tight (3.3Å)
Corrected (3.3Å)
1.0
0.8
0.6
0.4
0.2
0.0
DC
9Å
4.5Å
3Å
2.3Å
1.8Å

Supplementary Fig. 8 | Flow chart of single-particle analysis of the Omicron BA.2 S/RatACE2 complex. a, Representative one from 7,164 cryo-EM micrographs collected for the Omicron BA.2 S/RatACE2 complex. **b**, 2D class average images of the Omicron BA.2 S/RatACE2 complex. **c**, A workflow of cryo-EM image processing and reconstruction. **d**, The FSC curve for the density maps.

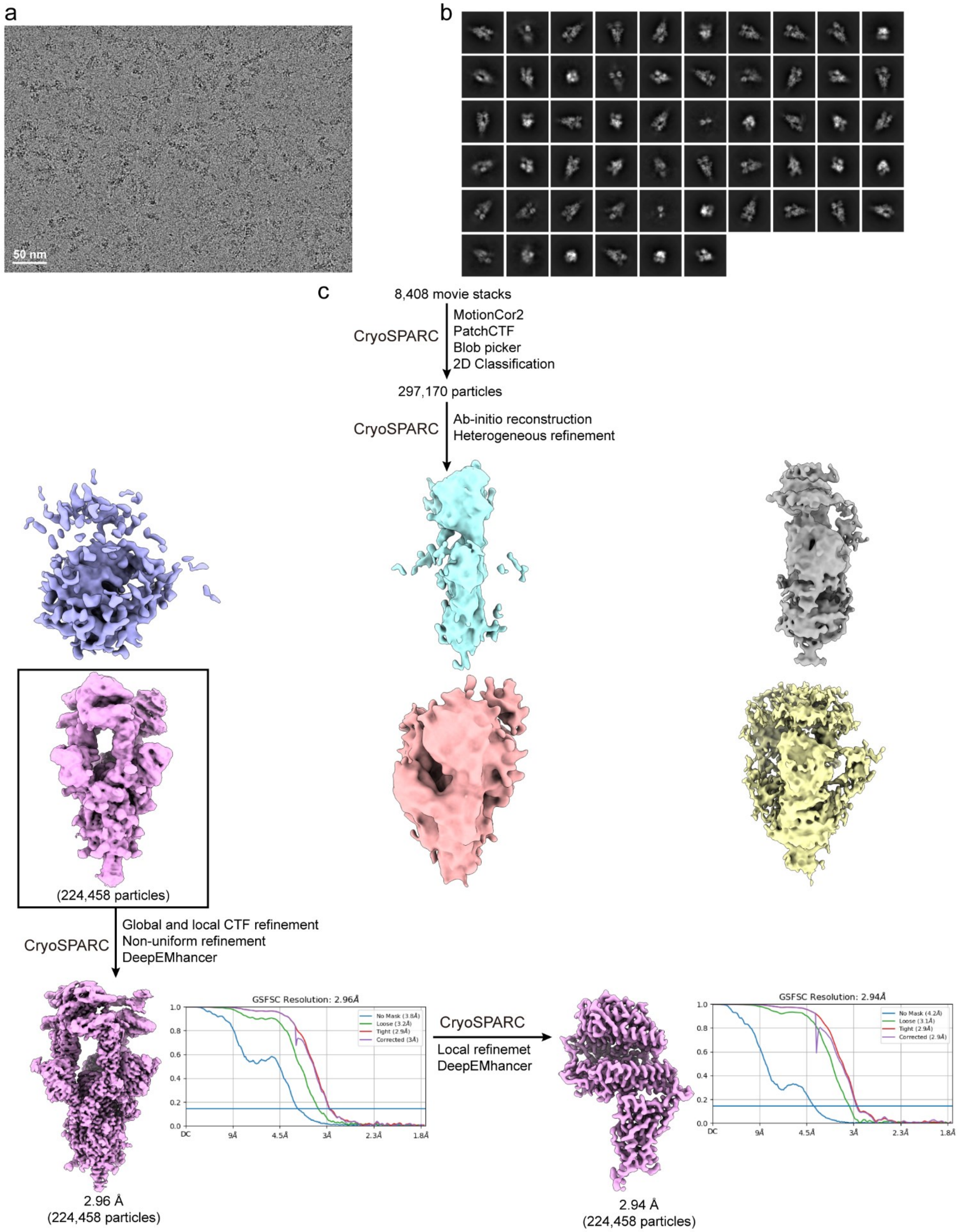

Supplementary Fig. 9 | Flow chart of single-particle analysis of the Omicron BA.2 S/ghACE2 complex. a, Representative one from 8,408 cryo-EM micrographs collected for the Omicron BA.2 S/ghACE2 complex. **b**, 2D class average images of the Omicron BA.2 S/ghACE2 complex. **c**, A workflow of cryo-EM image processing and reconstruction.

Supplementary Table 1. The immobilization and concentrations statistics of SPR assay to test the binding affinities between hACE2 and RBD of PT, BA.2, BA.2.12.1, BA.2.75 or BA.4/5.

Ligand	Immobilization quantity (units)	Concentrations of hACE2 (nM)	k_a (1/Ms)	k_d (1/s)	K_D (nM)	Average K_D (nM)	SD (nM)
prototype RBD	2702.5	200, 100, 50, 25, 12.5	1.9E+5	5.1E-3	27.4	23.8	2.6
			2.0E+5	4.4E-3	22.0		
			2.0E+5	4.5E-3	21.9		
BA.2 RBD	3588.9	200, 100, 50, 25, 12.5	1.7E+5	2.9E-3	17.5	14.6	2.1
			1.8E+5	2.3E-3	13.0		
			1.8E+5	2.4E-3	13.2		
BA.2.12.1 RBD	1570.2	200, 100, 50, 25, 12.5	1.7E+5	4.5E-3	26.1	27.4	0.9
			1.4E+5	4.0E-3	27.8		
			1.4E+5	4.0E-3	28.2		
BA.4/5 RBD	3240.6	200, 100, 50, 25, 12.5	1.1E+5	1.2E-3	11.5	9.0	1.7
			1.1E+5	8.8E-4	7.8		
			1.1E+5	8.9E-4	7.8		
BA.2.75 RBD	3399.9	50, 25, 12.5, 6.25, 3.125	1.4E+4	1.1E-5	7.8	7.5	0.2
			1.6E+4	1.2E-6	7.3		
			2.8E+5	2.1E-5	7.3		

Supplementary Table 2. Cryo-EM data collection, refinement and validation statistics.

	**BA.2 S/hACE2 (Global)**	**BA.2 S/hACE2 (Local)**	**BA.2.12.1 S/hACE2 (Global)**	**BA.2.12.1 S/hACE2 (Local)**	**BA.4/5 S/hACE2 (Global)**	**BA.4/5 S/hACE2 (Local)**
**Data collection and processing**						
Voltage (kV)	300	300	300	300	300	300
Electron exposure ($e^{-}/Å^2$)	50	50	50	50	50	50
Defocus range (μm)	-1.0 to -2.0	-1.0 to -2.0	-1.0 to -2.0	-1.0 to -2.0	-1.0 to -2.0	-1.0 to -2.0
Pixel size (Å)	0.85	0.85	0.85	0.85	0.88	0.88
Symmetry imposed	C1	C1	C1	C1	C1	C1
Final particle images (no.)	141,550	141,550	111,387	111,387	355,600	355,600
Map resolution (Å)	3.09	3.14	3.19	3.09	2.58	2.66
FSC threshold	0.143	0.143	0.143	0.143	0.143	0.143
**Refinement**						
R.m.s. deviations						
Bond lengths (Å)		0.006		0.005		0.003
Bond angles (°)		1.128		0.650		0.609
Validation						
Clashscore		7.31		4.30		2.58
Rotamer outliers (%)		3.61		0.29		0.58
Molprobity score		2.20		1.49		1.28
Ramachandran plot						
Favored (%)		94.54		95.93		96.44
Allowed (%)		4.45		4.07		3.43
Outliers (%)		1.02		0.00		0.13

	BA.2 S/ghACE2 (Global)	BA.2 S/ghACE2 (Local)	BA.2. S/mACE2 (Global)	BA.2 S/mACE2 (Local)	BA.2 S/RatACE2 (Global)	BA.2 S/RatACE2 (Local)
**Data collection and processing**						
Voltage (kV)	300	300	300	300	300	300
Electron exposure ($e^{-}/Å^{2}$)	50	50	50	50	50	50
Defocus range (μm)	-1.0 to -2.0	-1.0 to -2.0	-1.0 to -2.0	-1.0 to -2.0	-1.0 to -2.0	-1.0 to -2.0
Pixel size (Å)	0.88	0.88	0.67	0.67	0.88	0.88
Symmetry imposed	C1	C1	C1	C1	C1	C1
Final particle images (no.)	224,458	224,458	91,073	91,073	50,491	50,491
Map resolution (Å)	2.96	2.94	3.02	3.20	3.01	3.29
FSC threshold	0.143	0.143	0.143	0.143	0.143	0.143
**Refinement**						
R.m.s. deviations						
Bond lengths (Å)		0.005		0.004		0.002
Bond angles (°)		0.635		0.628		0.575
Validation						
Clashscore		3.85		6.66		6.11
Rotamer outliers (%)		0.15		0.29		0.14
Molprobity score		1.58		1.77		1.66
Ramachandran plot						
Favored (%)		93.92		94.03		95.30
Allowed (%)		6.08		5.46		4.70
Outliers (%)		0		0.51		0.00

Supplementary Table 3. X-ray data collection and refinement statistics.

	BA.2.75 RBD/hACE2
**Data collection**	
Space group	$P4_12_12$
Cell dimensions	
a, *b*, *c* (Å)	105.77, 105.77, 228.85
α, β, γ (°)	90.00, 90.00, 90.00
Resolution(Å)	50.00-2.90 (3.00-2.90)
Unique reflections	28751 (2859)
Completeness (%)	97.0 (98.2)
R_{sym}	0.162 (1.421)
$I/\sigma I$	12.1 (1.6)
$CC_{1/2}$ (%)	0.991 (0.633)
Redundancy	6.0 (6.2)
Wilson B ($Å^2$)	54.72
**Refinement**	
Resolution (Å)	39.04-2.90
No. reflections	27095
R_{work}/R_{free}	0.1967/0.2424
No. atoms	
Protein	6545
Ligand/ion	1
Water	0
B-factors ($Å^2$)	
Protein	55.8
Ligand/ion	33.8
Water	
R.M.S. deviations	
Bond lengths (Å)	0.004
Bond angles (°)	0.608
Ramchandran Statistics (%)	
Favored	96.44
Allowed	3.56
Disallowed	0

Values in parentheses are for the highest-resolution shell.

Supplementary Table 4. Amino acid residues comparison of BA.2, BA.2.12.1, BA.2.75 and BA.4/5 RBDs interacting with hACE2.

hACE2	BA.2 RBD	BA.2.12.1 RBD	BA.2.75 RBD	BA.4/5 RBD
S19	A475 (2), N477 (7, **1**)	A475 (3, **1**), N477 (4)	A475 (3, **1**), G476 (3), N477 (12, **1**)	N477 (9, **1**)
Q24	A475 (3), G476 (2), N477 (1), N487 (9, **1**)	A475 (4), G476 (1), N477 (1), N487 (9)	A475 (4), G476 (4), N477 (2), N487 (15), Y489 (1)	A475 (7), G476 (3), N477 (1), N487 (6, **1**)
T27	F456 (4), Y473 (1), A475 (1), Y489 (4)	F456 (6), Y473 (1), A475 (1), Y489 (4)	F456 (8), Y473 (1), Y489 (5)	F456 (5), Y473 (1), A475 (3), Y489 (5)
F28	Y489 (4)	Y489 (6)	Y489 (6)	Y489 (5)
D30	L455 (1)	L455 (1), F456 (2)	F456 (4)	L455 (1), F456 (1)
K31	F456 (1), Y489 (6), R493 (2)	F456 (2), Y489 (4), R493 (2)	F456 (4), Y489 (5), Q493 (8)	F456 (1), Y489 (2), F490 (2), Q493 (7, **1**)
H34	Y453 (2), R493 (10), S494 (6)	Y453 (6), R493 (11), S494 (5, **1**)	N417 (2), Y453 (8), L455 (6)	Y453 (5, **1**), L455 (8), Q493 (14), S494 (6)
E35	R493 (6, **1**)	R493 (4, **1**)	-	Q493 (3)
E37	-	-	H505 (1)	-
D38	Y449 (5, **2**), R498 (4, **1**), Y501 (1)	Y449 (5, **1**), R498 (4, **1**), Y501 (1)	Y449 (9, **1**), R498 (6, **1**), Y501 (1)	Y449 (4, **2**), R498 (4, **1**)
Y41	R498 (2), T500 (6, **1**), Y501 (18)	R498 (3), T500 (8, **1**), Y501 (15)	R498 (3), T500 (7, **1**), Y501 (13)	R498 (4), T500 (7, **1**), Y501 (13)
Q42	Y449 (1), R498 (5, **1**)	Y449 (1,), R498 (10)	Y449 (2,**1**), R498 (8)	Y449 (1), R498 (6)
L45	-	-	T500 (1)	-
L79	F486 (1)	F486 (1)	F486 (2)	V486 (1)
M82	F486 (8)	F486 (8)	F486 (9)	V486 (2), N487 (4)
Y83	F486 (8), N487 (4, **1**), Y489 (1)	F486 (8), N487 (5, **1**), Y489 (1)	F486 (9), N487 (7, **1**), Y489 (1)	N487 (3, **1**), Y489 (1)
N330	-	-	T500 (6)	-
K353	Y501 (18), G502 (3, **1**), H505 (18)	Y501 (19), G502 (3, **1**), H505 (19)	Y501 (22), G502 (3, **1**), H505 (24)	Y501 (16), G502 (3, **1**), H505 (18)
G354	G502 (6), H505 (1)	G502 (6), H505 (1)	G502 (7), H505 (2)	G502 (6), H505 (1)
D355	T500 (10, **1**)	T500 (9, **1**)	T500 (7), G502 (1)	T500 (7, **1**)
R357	T500 (2, **1**)	T500 (3, **1**)	T500 (3)	T500 (2)
Total	194, **12**	207, **10**	255, **8**	198, **11**

The numbers in parentheses of BA.2 RBD, BA.2.12.1 RBD and BA.4/5 RBD residues represent the number of Van der Waals' contacts between the indicated residues with hACE2. The numbers with underline suggest numbers of potential H-bonds between the pairs of residues. Van der Waals' contacts were analyzed at a cutoff of 4.5 Å, and H-bonds were calculated at a cutoff of 3.5 Å. Interacting residues in each row that are not shared by all the variants are colored red.

Supplementary Table 5. The immobilization and concentrations statistics of SPR assay to test the binding affinities between hACE2 and RBDs of BA.4/5, BA.2 and BA.4/5-RBD-based mutants.

Ligand	Immobilization quantity (units)	Concentrations of hACE2 (nM)	k_a (1/Ms)	k_d (1/s)	K_D (nM)	Average K_D (nM)	SD (nM)
BA.4/5 RBD	6483.0	200, 100, 50, 25, 12.5	8.1E+4	1.2E-3	15.0	12.9	1.8
			1.2E+5	1.9E-3	12.6		
			8.6E+4	1.2E-3	13.8		
			7.2E+4	1.4E-3	10.1		
BA.2 RBD	5244.5	200, 100, 50, 25, 12.5	1.2E+5	2.4E-3	20.5	19.5	1.7
			1.1E+5	1.9E-3	17.8		
			1.4E+5	2.5E-3	18.0		
			1.1E+5	2.8E-3	21.8		
BA.4/5-V486F RBD	4015.4	200, 100, 50, 25, 12.5	1.3E+5	4.3E-3	3.2	4.0	0.7
			1.1E+5	4.4E-3	3.8		
			1.2E+5	4.4E-3	3.8		
			1.4E+5	4.3E-3	5.1		
BA.4/5-R452L RBD	3091.9	200, 100, 50, 25, 12.5	6.9E+4	1.1E-3	14.7	14.1	0.6
			6.0E+4	7.9E-4	13.2		
			5.4E+4	8.4E-4	14.4		
			1.3E+5	3.2E-3	14.1		
BA.4/5-Q493R RBD	3323.3	200, 100, 50, 25, 12.5	6.2E+5	7.4E-5	137.0	127.0	14.5
			5.3E+5	8.0E-5	135.0		
			5.1E+5	1.1E-6	102.0		
			5.2E+5	6.4E-5	134.0		

Supplementary Table 6. The immobilization and concentrations statistics of SPR assay to test the binding affinities of different ACE2 orthologs with BA.1, BA.2, BA.2-R493Q and BA.4/5 RBDs.

Immobilized ligand	Immobilization quantity (units)	Analyte	Concentrations of RBD (nM)	k_a (1/Ms)	k_d (1/s)	K_D (nM)	Average K_D (nM)	SD （nM）
Rabbit ACE2	7789.6	BA.1 RBD	800, 400, 200, 100, 50	2.0E+6	2.2E-2	60.0	43.7	11.5
				1.7E+6	1.0E-2	35.2		
				1.5E+6	1.5E-2	36.0		
		BA.2 RBD	200, 100, 50, 25, 12.5	2.4E+5	3.7E-2	48.0	59.3	12.2
				2.5E+5	3.8E-2	76.3		
				2.9E+5	2.0E-2	53.8		
		BA.2-R493Q RBD	50, 25, 12.5, 6.25, 3.125	2.7E+5	1.8E-3	7.5	5.9	1.6
				3.0E+5	4.6E-2	6.4		
				3.2E+5	3.5E-2	3.8		
		BA.4/5 RBD	50, 25, 12.5, 6.25, 3.125	1.9E+5	6.2E-4	2.9	3.0	0.2
				6.0E+4	5.4E-4	3.3		
				7.0E+4	6.8E-4	2.7		
Mouse ACE2	10763.3	BA.1 RBD	400, 200, 100, 50, 25	4.6E+5	3.8E-2	35.3	32.7	3.6
				6.8E+5	4.4E-2	35.1		
				7.4E+5	6.7E-2	27.6		
		BA.2 RBD	200, 100, 50, 25, 12.5	2.5E+6	4.5E-2	9.5	13.3	3.1
				2.2E+6	5.5E-2	17.1		
				2.4E+6	4.4E-2	13.2		
		BA.2-R493Q RBD	800, 400, 200, 100, 50	3.5E+4	4.7E-2	67.1	78.2	9.8
				3.0E+4	4.7E-2	91.0		
				3.9E+4	4.7E-2	76.4		
		BA.4/5 RBD	400, 200, 100, 50, 25	1.6E+5	4.7E-2	42.7	42.7	1.5
				1.8E+5	4.7E-2	40.9		
				1.3E+5	4.7E-2	44.4		

Rat ACE2	8855.4	BA.1 RBD	6400, 3200, 1600, 800, 400	3.0E+4	1.1E-1	3458.8	3414.8	36.7
				3.1E+4	1.1E-1	3368.8		
				3.2E+4	1.1E-1	3416.8		
		BA.2 RBD	6400, 3200, 1600, 800, 400	5.7E+3	9.9E-3	1888.2	2037.4	116.0
				6.5E+3	9.9E-3	2052.7		
				5.5E+3	8.1E-3	2171.2		
		BA.2-R493Q RBD	6400, 3200, 1600, 800, 400	1.6E+4	1.2E-1	4584.9	4068.8	366.6
				1.8E+4	1.5E-1	3852.9		
				2.1E+4	2.1E-1	3768.6		
		BA.4/5 RBD	6400, 3200, 1600, 800, 400	2.1E+4	2.1E-2	1061.2	1024.4	26.3
				2.0E+4	2.8E-2	1010.8		
				3.9E+4	3.2E-2	1001.1		
Golden Hamster ACE2	12612.6	BA.1 RBD	1600, 800, 400, 200, 100	9.4E+4	2.6E-2	277.0	267.7	27.7
				1.1E+5	6.1E-2	230.0		
				1.8E+4	5.3E-3	296.0		
		BA.2 RBD	800, 400, 200, 100, 50	2.5E+6	8.6E-1	174.4	149.1	30.5
				3.0E+6	10.0E-1	106.2		
				2.0E+6	7.5E-1	166.7		
		BA.2-R493Q RBD	6400, 3200, 1600, 800, 400	1.9E+4	2.7E-2	1061.5	1219.0	171.9
				1.8E+4	2.4E-2	1458.1		
				1.9E+4	9.5E-3	1137.5		
		BA.4/5 RBD	800, 400, 200, 100, 50	2.6E+4	1.4E-3	117.7	133.4	22.7
				1.3E+4	1.0E-2	117.1		
				1.6E+4	1.7E-2	165.6		
Cat ACE2	11219.9	BA.1 RBD	800, 400, 200, 100, 50	2.8E+6	9.9E-1	60.8	68.2	7.5
				4.2E+6	3.1E-2	78.5		
				2.0E+6	1.7E-2	65.2		
		BA.2 RBD	800, 400, 200, 100, 50	1.8E+5	1.4E-2	133.0	139.5	13.1
				2.0E+5	3.6E-2	127.7		
				1.0E+5	2.9E-2	157.8		
		BA.2-R493Q RBD	1600, 800, 400, 200, 100	3.5E+5	1.1E-1	208.9	285.7	54.5
				4.5E+5	1.2E-1	318.6		

				3.8E+5	1.4E-1	329.5		
		BA.4/5 RBD	800, 400, 200, 100, 50	4.7E+5	6.9E-2	149.2	154.7	7.9
				5.7E+5	7.1E-2	148.9		
				3.8E+5	7.9E-2	165.9		
Dog ACE2	8102.1	BA.1 RBD	1600, 800, 400, 200, 100	2.1E+5	1.6E-1	476.4	533.6	44.7
				4.2E+4	1.0E-2	539.0		
				2.9E+5	6.5E-2	585.4		
		BA.2 RBD	1600, 800, 400, 200, 100	3.3E+6	1.5E-1	221.6	231.1	18.7
				2.8E+6	1.6E-1	257.2		
				2.3E+6	3.4E-1	214.6		
		BA.2-R493Q RBD	3200, 1600, 800, 400, 200	1.5E+5	6.0E-1	766.8	962.5	141.2
				1.5E+6	7.1E-1	1026.2		
				1.8E+6	5.9E-1	1094.5		
		BA.4/5 RBD	1600, 800, 400, 200, 100	6.0E+5	2.7E-2	211.2	185.6	28.7
				4.2E+6	3.5E-2	145.5		
				7.8E+6	2.3E-2	200.0		
Horse ACE2	8755.8	BA.1 RBD	12800, 6400, 3200, 1600, 800	3.0E+4	2.3E-1	7436.7	7980.5	540.6
				3.2E+4	2.5E-1	7787.2		
				3.0E+4	2.6E-1	8717.8		
		BA.2 RBD	40000, 20000, 10000, 5000, 2500	9.8E+3	3.8E-1	38207.5	40522.3	2888.7
				9.6E+3	3.7E-1	38764.4		
				8.4E+3	3.7E-1	44594.8		
		BA.2-R493Q RBD	2000, 1000, 500, 250, 125	9.4E+4	3.6E-1	3806.4	4094.5	384.3
				7.7E+4	3.6E-1	4637.6		
				8.0E+4	3.1E-1	3839.6		
		BA.4/5 RBD	1000, 500, 250, 125, 62.5	4.5E+4	2.0E-2	455.2	453.3	5.9
				4.6E+4	2.1E-2	445.3		
				4.5E+4	2.1E-2	459.4		
Pig ACE2	8089.0	BA.1 RBD	800, 400, 200, 100, 50	1.6E+5	9.2E-2	566.2	570.7	18.1
				1.6E+5	8.8E-2	551.2		
				1.6E+5	9.3E-2	594.7		
		BA.2 RBD	1000, 500, 250, 125, 62.5	1.4E+5	9.8E-2	718.7	678.0	29.0

				1.5E+5	9.6E-2	653.7		
				1.5E+5	9.8E-2	661.6		
		BA.2-R493Q RBD	400, 200, 100, 50, 25	4.3E+5	1.2E-2	27.9	25.2	2.0
				4.5E+5	1.1E-2	23.2		
				4.4E+5	1.1E-2	24.5		
		BA.4/5 RBD	100, 50, 25, 12.5, 6.25	3.6E+5	2.2E-3	6.1	7.1	0.8
				3.6E+5	2.6E-3	7.2		
				3.2E+5	2.5E-3	8.0		
Goat ACE2	6604.8	BA.1 RBD	400, 200, 100, 50, 25	3.3E+5	5.7E-2	173.7	180.4	5.7
				5.1E+5	9.1E-2	179.8		
				7.1E+5	1.3E-1	187.6		
		BA.2 RBD	400, 200, 100, 50, 25	3.4E+5	3.8E-2	110.8	106.1	3.7
				3.5E+5	3.6E-2	101.9		
				4.2E+5	4.5E-2	105.6		
		BA.2-R493Q RBD	400, 200, 100, 50, 25	1.7E+5	2.0E-3	11.5	11.3	0.6
				1.4E+5	1.4E-3	10.4		
				1.9E+5	2.2E-3	11.9		
		BA.4/5 RBD	400, 200, 100, 50, 25	8.1E+4	2.3E-3	29.1	26.8	1.6
				6.5E+4	1.7E-3	26.2		
				6.0E+4	1.5E-3	25.3		
Sheep ACE2	7141.0	BA.1 RBD	200, 100, 50, 25, 12.5	3.9E+5	2.4E-2	62.0	66.8	3.9
				6.6E+5	4.4E-2	66.8		
				2.0E+6	1.5E-1	71.5		
		BA.2 RBD	400, 200, 100, 50, 25	2.1E+5	7.4E-3	35.4	34.0	1.2
				2.0E+5	6.5E-3	32.5		
				2.0E+5	6.8E-3	34.2		
		BA.2-R493Q RBD	200, 100, 50, 25, 12.5	3.2E+5	9.6E-4	3.0	3.1	0.1
				2.6E+5	8.2E-4	3.1		
				3.7E+5	1.2E-3	3.3		
		BA.4/5 RBD	400, 200, 100, 50, 25	1.3E+5	1.4E-3	10.9	9.4	1.1
				1.2E+5	9.9E-4	8.4		
				1.1E+5	1.0E-3	8.9		

Supplementary Table 7. Amino acid residues comparison of BA.2 RBD interacting with mACE2, ratACE2 and ghACE2.

BA.2 RBD	mACE2	RatACE2	ghACE2
Y449	D38 (4), Q42 (1)	Q42 (1)	D38 (4, **1**)
Y453	Q34 (2)	Q34 (3)	Q34 (3)
L455	Q34 (2)	-	Q34 (2)
F456	T27 (5), N30 (2), N31 (2)	S27 (9)	T27 (5), D30 (1), K31 (2)
Y473	-	-	T27 (1)
A475	N24 (3), T27 (1)	K24 (4)	Q24 (3)
G476	N24 (2)	K24 (1)	Q24 (1)
F486	T79 (1), F83 (2)	I79 (2), N82 (2), F83 (4)	L79 (4), N82 (3), Y83 (5), NAG708 (4)
N487	N24 (2)	K24 (5), F83 (1)	Q24 (7, **1**), Y83 (4, **1**)
Y489	T27 (2), F28 (4), N31 (3)	S27 (4), F28 (4), K31 (4)	T27 (4), F28 (4), K31 (5), Y83 (1),
R493	N31 (7, **2**), Q34 (16), E35 (2)	K31 (3), Q34 (11, **1**),	K31 (2), Q34 (14)
S494	Q34 (1)	Q34 (2)	Q34 (3, **1**)
Y495	-	D38 (1)	K353 (1)
R498	D38 (1), Y41 (4), Q42 (3)	Y41 (3), Q42 (7), L45 (1)	D38 (5, **2**), Y41 (4), Q42 (5)
T500	Y41 (7, **1**), N330 (1), D355 (7), R357 (3)	Y41 (4, **1**), N330 (1), D355 (3), R357 (2)	Y41 (7, **1**), N330 (1), D355 (9), R357 (3),
Y501	Y41 (10), H353 (23), G354 (1)	D38 (1), Y41 (16), H353 (22, **1**), G354 (1),	Y41 (12), K353 (21, **1**),
G502	H353 (3, **1**), G354 (6)	H353 (3, **1**), G354 (5)	K353 (3, **1**), G354 (6)
V503	-	P325 (1)	-
H505	H353 (20)	H353 (7)	K353 (12)
Total	153, **4**	138, **4**	171, **9**

The numbers in parentheses of mACE2, ratACE2 and ghACE2 residues represent the number of Van der Waals' contacts between the indicated residues with BA.2 RBD. The numbers with underline suggest numbers of potential H-bonds between the pairs of residues. Van der Waals' contacts were analyzed at a cutoff of 4.5 Å, and H-bonds were calculated at a cutoff of 3.5 Å.
