# Supplementary material for: Structural basis for receptor binding and broader interspecies receptor recognition of currently circulating Omicron sub-variants
Source: Nat Commun. 2023 Jul 21;14:4405. doi: 10.1038/s41467-023-39942-z (PMC10362042; doi:10.1038/s41467-023-39942-z)
Supplement: Supplementary file 3 — Reporting Summary [file 41467_2023_39942_MOESM3_ESM.pdf]

nature portfolio

Corresponding author(s): George F. Gao

Last updated by author(s): Jun 13, 2023

Reporting Summary

Nature Portfolio wishes to improve the reproducibility of the work that we publish. This form provides structure for consistency and transparency in reporting. For further information on Nature Portfolio policies, see our Editorial Policies and the Editorial Policy Checklist.

Statistics

For all statistical analyses, confirm that the following items are present in the figure legend, table legend, main text, or Methods section.

n/a	Confirmed	
☐	☒	The exact sample size (*n*) for each experimental group/condition, given as a discrete number and unit of measurement
☐	☒	A statement on whether measurements were taken from distinct samples or whether the same sample was measured repeatedly
☒	☐	The statistical test(s) used AND whether they are one- or two-sided<br>*Only common tests should be described solely by name; describe more complex techniques in the Methods section.*
☒	☐	A description of all covariates tested
☒	☐	A description of any assumptions or corrections, such as tests of normality and adjustment for multiple comparisons
☐	☒	A full description of the statistical parameters including central tendency (e.g. means) or other basic estimates (e.g. regression coefficient) AND variation (e.g. standard deviation) or associated estimates of uncertainty (e.g. confidence intervals)
☒	☐	For null hypothesis testing, the test statistic (e.g. *F*, *t*, *r*) with confidence intervals, effect sizes, degrees of freedom and *P* value noted<br>*Give P values as exact values whenever suitable.*
☒	☐	For Bayesian analysis, information on the choice of priors and Markov chain Monte Carlo settings
☒	☐	For hierarchical and complex designs, identification of the appropriate level for tests and full reporting of outcomes
☒	☐	Estimates of effect sizes (e.g. Cohen's *d*, Pearson's *r*), indicating how they were calculated

Our web collection on statistics for biologists contains articles on many of the points above.

Software and code

Policy information about availability of computer code

Data collection	CryoEM data were collected using Thermo Scientific EPU. X-ray diffraction data were collected under cryogenic conditions (100K) at Shanghai Synchrotron Radiation Facility (SSRF). The BA.2.75 RBD/hACE2 dataset was collected at BL02U1.For the pseudovirus neutralization assay,a CQ1 confocal image cytometer (Yokogawa) was used for imaging and fluorescent cells determination. Biacore 8K (Cytiva) was used to collect the binding profile data for SPR analysis. BD FACS Canto? Flow Cytometer (BD Biosciences) was used for flow cytometry assay.
Data analysis	Cryo-EM data processing was performed using following packages: MotionCor2 v.1.4.2, RELION-3.1, cryoSPARC v3.3.1, DeepEMhancer v0.14. The X-ray data were indexed, integrated, and scaled with HKL2000. The crystal structure was then determined by the molecular replacement method using Phaser. The model building, refinement and evaluation of stereochemical qualities for cryo-EM or X-ray structures were performed using PHENIX v.1.19.2, Coot v.0.9.8 and Molprobity. PyMOL v.2.4 and UCSF Chimera v.1.14 and ChimeraX v.1.3 were used to generate the structural figures. GraphPad Prism 8 and Biacore Insight Evaluation software v.3.0 were used to do data analysis and make some figures.FlowJo v10.8 Software was used for FACS analysis.

For manuscripts utilizing custom algorithms or software that are central to the research but not yet described in published literature, software must be made available to editors and reviewers. We strongly encourage code deposition in a community repository (e.g. GitHub). See the Nature Portfolio guidelines for submitting code & software for further information.

Data

Policy information about availability of data

All manuscripts must include a data availability statement. This statement should provide the following information, where applicable:

- Accession codes, unique identifiers, or web links for publicly available datasets
- A description of any restrictions on data availability
- For clinical datasets or third party data, please ensure that the statement adheres to our policy

The atomic structure coordinates were deposited in the RCSB Protein Data Bank (PDB) under the accession codes 7YJ3, 7YHW, 8H06, 7YVU, 8GRY, 7YV8 and 8H5C, all of which were also deposited in China National Microbiology Data Center (NMDC) with accession numbers NMDCS0000020, NMDCS0000021, NMDCS0000025, NMDCS0000023, NMDCS0000024, NMDCS0000022 and NMDCS0000026. Electron microscopy maps were deposited in the Electron Microscopy Data Bank under accession codes EMD-33870, EMD-33841, EMD-34409, EMD-34138, EMD-34217, EMD-34120, EMD-34494, EMD-34498, EMD-34499, EMD-34509, EMD-34510 and EMD-34506. Other structures for analysis, including 7KNB and 6LZG, were obtained from the PDB. Source data are provided with this paper.

Human research participants

Policy information about studies involving human research participants and Sex and Gender in Research.

Reporting on sex and gender	N/A
Population characteristics	N/A
Recruitment	N/A
Ethics oversight	N/A

Note that full information on the approval of the study protocol must also be provided in the manuscript.

Field-specific reporting

Please select the one below that is the best fit for your research. If you are not sure, read the appropriate sections before making your selection.

☒ Life sciences ☐ Behavioural & social sciences ☐ Ecological, evolutionary & environmental sciences

For a reference copy of the document with all sections, see nature.com/documents/nr-reporting-summary-flat.pdf

Life sciences study design

All studies must disclose on these points even when the disclosure is negative.

Sample size	The amount of cryo-EM micrographs collected was based on the cryo-EM time allocation and previous knowledge estimating that the size is sufficient to generate a high-resolution density (Zhao, Z. et al. Omicron SARS-CoV-2 mutations stabilize spike up-RBD conformation and lead to a non-RBM-binding monoclonal antibody escape. Nat. Commun. 13, 4958 (2022). The details of cryo-EM datasets are given in Methods and Supplementary Information. No sample size calculation was performed. A total of 10,317 images of the Omicron BA.2 S/hACE2 complex, 6,768 images of Omicron BA.2.12.1 S/hACE2 complex, 6,282 images of Omicron BA.4/5 S/hACE2 complex, 6,628 images of the BA.2 S/mACE2 complex, 7,164 images of the BA.2 S/RatACE2 complex and 8,408 images of the BA.2 S/ghACE2 complex were collected using 300 kV Titan Krios electron microscope. These samples turned out to be sufficient for us to achieve the resolution of the cryo-EM reconstruction to a near-atomic level.
Data exclusions	No data were excluded from analyses.
Replication	SPR was repeated three or four times with similar results. Flow cytometry analysis for receptor binding was performed three times with similar results. VSV-based pseudovirus infection assay was conducted twice with six duplicates each time with similar results.
Randomization	Randomization is not applicable to the study with automated movie acquisition and data analysis conducted using reference-free (unbiased) class averaging.
Blinding	Blinding is not relevant for this study because there was no specific grouping in data collection and analysis.

Reporting for specific materials, systems and methods

We require information from authors about some types of materials, experimental systems and methods used in many studies. Here, indicate whether each material, system or method listed is relevant to your study. If you are not sure if a list item applies to your research, read the appropriate section before selecting a response.

Materials & experimental systems

n/a	Involved in the study
☐	☒ Antibodies
☐	☒ Eukaryotic cell lines
☒	☐ Palaeontology and archaeology
☒	☐ Animals and other organisms
☒	☐ Clinical data
☒	☐ Dual use research of concern

Methods

n/a	Involved in the study
☒	☐ ChIP-seq
☐	☒ Flow cytometry
☒	☐ MRI-based neuroimaging

Antibodies

Antibodies used

Anti-His/APC fluorescence antibody (diluted at a ratio of 1:500, Miltenyi Biotec, 130-119-820) was used in the flow cytometry assay for receptor binding. Anti-VSV-G antibody (10 µg/mL), produced by I1-Hybridoma (ATCC, CRL-2700), was used during the pseudovirus production.

Validation

Anti-His/APC fluorescence antibody is a mouse monoclonal antibody against His tag conjugated to APC. It is commercially available, and the validation is listed on its website:
https://www.miltenyibiotec.com/US-en/products/his-antibody-gg11-8f3-5-1.html#conjugate=apc:size=100-tests-in-200-ul

Anti-VSV-G antibody is a mouse monoclonal antibody reacting with the major surface glycoprotein (G-protein) of vesicular stomatitis virus, Indiana Serotype. It is generated by I1-Hybridoma, which is a hybridoma: b lymphocyte cell line isolated from an adult mouse with vesicular stomatitis. I1-Hybridoma is commercially available, and the validation is listed on its website:
https://www.atcc.org/products/crl-2700

Eukaryotic cell lines

Policy information about cell lines and Sex and Gender in Research

Cell line source(s)

HEK293F cells (Cat# 11625-019) was bought from Gibco. HEK293T (Cat# CRL-3216) , BHK-21 (Cat# CCL-10) and Vero cells (Cat# CCL-81) were bought from ATCC.

Authentication

No cell authentication method was used.

Mycoplasma contamination

All cell lines tested negative for mycoplasma.

Commonly misidentified lines
(See ICLAC register)

No misidentified lines were used.

Flow Cytometry

Plots

Confirm that:

☒ The axis labels state the marker and fluorochrome used (e.g. CD4-FITC).

☒ The axis scales are clearly visible. Include numbers along axes only for bottom left plot of group (a 'group' is an analysis of identical markers).

☒ All plots are contour plots with outliers or pseudocolor plots.

☒ A numerical value for number of cells or percentage (with statistics) is provided.

Methodology

Sample preparation

The plasmids encoding 29 full-length ACE2 orthologs (human, monkey, grivet, chimpanzee, gorilla, rabbit, mouse, rat, guinea pig, golden hamster, Malayan pangolin, cat, dog, horse, pig, fox, civet, mink, goat, sheep, camel, alpaca, bovine, little brown bat, fulvous fruit bat, greater horseshoe bat, Chinese horseshoe bat, least horseshoe bat and lesser hedgehog tenrec) fused with eGFP at the C-terminal were transfected into BHK-21 cells using PEI at the mass ratio of 1:3. Solutions containing the RBD (2 µg/mL) of BA.1, BA.2 or BA.4/5 were incubated with 29 ACE2 orthologs (including hACE2)-expressing BHK-21 cells at 37 degrees ?for 1h, respectively. Subsequently, cells were washed with PBS thrice and stained with Anti-His (APC) antibody (Miltenyi, Cat# 130-119-820) for 1 h. ACE2-transfected BHK-21 cells incubated with the N-terminal domain (NTD) of SARS-CoV-2 PT and BHK-21 cells transfected using CD26 fused with eGFP were used as negative controls.

Instrument

BD FACS Canto? Flow Cytometer (BD Biosciences)

Software	The data were analyzed by FlowJo v10.8 Software.
Cell population abundance	Each samples collected 10000 cells.
Gating strategy	Untransfected cells were used to define the boundary of positive and negative staining.

☒ Tick this box to confirm that a figure exemplifying the gating strategy is provided in the Supplementary Information.
